# Association of IL-1beta gene polymorphism with cachexia from locally advanced gastric cancer

**DOI:** 10.1186/1471-2407-7-45

**Published:** 2007-03-14

**Authors:** Dianliang Zhang, Hongmei Zheng, Yanbing Zhou, Xingming Tang, Baojun Yu, Jieshou Li

**Affiliations:** 1 Department of General Surgery, Affiliated Hospital of Qingdao University Medical College, Qingdao 266003, P. R. China; 2 Research Institute of General Surgery, Jinlin Hospital, Nanjing University, Nanjing 210093, P. R. China

## Abstract

**Background:**

IL-1beta has been implicated in inflammatory episode. In view of the inflammatory nature of cancer cachexia, we determined the predictive value of IL-1B-31 T/C, -511 C/T, +3954 C/T and IL-1RN VNTR gene polymorphisms on the occurrence of cachexia associated with locally advanced gastric cancer.

**Methods:**

The study included 214 patients and 230 healthy volunteers. Genomic DNA was prepared from peripheral blood leukocytes. Genotypes and allele frequencies were determined in patients and healthy controls using restriction fragment length polymorphism analysis of polymerase chain reaction products.

**Results:**

The overall frequencies of IL-1B-31 T, -511 T, +3954 T and IL-1RN VNTR alleles in patients with locally advanced gastric cancer were all comparable with those in controls. No significant differences were found in the distribution of IL-1B-31 T, -511 T and IL-1RN VNTR between patients with cachexia and without. Patients with cachexia showed a significantly higher prevalence of IL-1B+3954 T allele than those without (*P *= 0.018). In a logistic regression analysis adjusted for actual weight, carcinoma location and stage, the IL-1B+3954 CT genotype was associated with an odds ratio of 2.512 (95% CI, 1.180 – 5.347) for cachexia.

**Conclusion:**

The IL-1B+3954 T allele is a major risk for cachexia from locally gastric cancer. Genetic factors studied are not likely to play an important role in the determination of susceptibility to locally advanced gastric cancer.

## Background

Cancer cachexia is a syndrome characterized by a marked weight loss, anorexia, asthenia and anaemia, accounting for at least 20% of deaths in neoplastic patients [1]. Unlike starvation, which depletes fat stores form adipose tissue while conserving protein from the wasting of skeletal muscle, in cachexia neither fat nor protein is spared. Progressive weight loss is a common feature of cancer and is responsible not only for a poor quality of life and poor response to chemotherapy, but also short survival time irrespective of tumor mass or the presence of metastases, and it also interferes with cancer therapy [2]. Although the mechanism of cancer cachexia remains largely unresolved, several key mediators have been identified. One set is proinflammatory cytokines [3], including TNF-α, IL-1β, IL-6 and IFN-γ [4,5]. In humans, there is increasing evidence that the host's cytokine response is genetically determined. Polymorphic gene sequences of certain cytokines could be potential markers of susceptibility and clinical outcome in different human infectious diseases. However, to the best of our knowledge, no study has addressed the correlation of cytokine polymorphisms of IL-1β gene with the likelihood of cachexia from locally advanced gastric cancer.

IL-1β is a potent proinflammatory cytokine released by macrophages in systemic inflammatory responses. It not only has important biologic effect but also regulates inflammatory reaction and immune response through promoting other cytokines expressions, such as IL-6 and IL-12. Studies have documented constitutive IL-1β protein production in human and animal cancer cell lines including sarcomas and ovarian and transitional cell carcinomas. Solid tumors in which IL-1β has been shown to be up regulated include breast, colon, lung, head and neck cancers, and melanomas, and patients with IL-1β producing tumors have generally bad prognoses. It has also been reported that this cytokine was the factor responsible for enhanced protein degradation in rats, and repeated administration of IL-1 to rats induced anorexia and weight loss [6]. In vivo, the potentially injurious proinflammatory effects of IL-1β are counterbalanced by the action of the interleukin-1 receptor antagonist (IL-1RN) [7]. Extensive evidence indicates that the biologic function of secreted IL-1RN is to competitively inhibit the binding of circulating IL-1β to cell-surface receptors [8,9], and IL-1RN levels increase late during the course of an inflammatory event to terminate acute inflammation [7].

The production of IL-1β and IL-1RN are depended on several factors, there is increasing evidence that the genetic factors plays an important role. The IL-1B and the IL-1RN genes are located on chromosome 2q14, within a 360 kb region. In the IL1B gene there are two diallelic polymorphisms at positions -511, -31 in the promoter region and at position + 3954 in the fifth exon. The IL-1RN gene has a penta-allelic polymorphic site in intron 2 containing variable numbers of an 86 bp tandem repeat sequence. In recent years, in vitro and in vivo studies have indicated that IL-1B -511 T and +3954 T alleles enhance IL-1β and IL-1RN productions and the circulating levels of the two cytokines in humans [10,11]. It has been reported that proinflammatory genotypes of the interleukin-1 loci were associated with a significantly increased risk of a chronic hypochlorhydric response to H. pylori infection and gastric cancer in a Caucasian population, presumably by altering IL-1β levels in the stomach [12,13], but an influence of IL-1β gene polymorphisms on susceptibility of cachexia from gastric cancer has not been evaluated. In view of the roles of IL-1β played in cachexia, we postulated that its polymorphisms might have some association with the development of cancer cachexia.

The causes of cachexia are thought to be multifactorial but primarily related to hypercatabolism and loss of appetite due to circulating tumor-related substances and cytokines [14,15]. Working from assumption of substantial inflammatory contribution to cancer cachexia, we hypothesized that IL-1B-31 T/C, -511 C/T, +3954 C/T and IL-1RN gene polymorphisms have some relationship with the development of cachexia associated with locally advanced gastric cancer. We also determined whether the gene polymorphisms studied were associated with the susceptibility to locally advanced gastric cancer.

## Methods

### Study population

The protocol was approved by the local Ethics Committee, and informed consent was obtained from each of the patient or a close relative. All of the consecutive patients with locally advanced gastric cancer from June 1, 2004 to June 1, 2006 were prospectively considered. To be eligible for enrollment, all of the subjects had to be Chinese Han Population. The exclusion criteria were the following: (1) anorexia nervosa; (2) pyloric obstruction; (3) major gastrointestinal disease, chronic renal failure, diabetes and HIV; (4) hepatic, peritoneal, pelvic and distant metastasis; (5) consanguineous mating. Immediately after admission and before any surgical or medical procedure was begun, patients were investigated for the presence of anorexia, weight loss, smoking and drinking water status. The controls were recruited from hospital attendees in two centers with no family history of gastric cancer. They were matched with patients for age and sex, without any malignant diseases and infectious disorders.

Information on smoking and drinking water status was obtained by interview. We did not obtain dietary histories, such as details of meat or fish consumption. We defined "current" as persons who were current smokers, "ex-" as those who had smoked in the past, and "never" as those who had never smoked at any time in their life.

Weight loss during the proceeding 6 months was expressed as a percentage of the usual weight and patients were divided into 2 groups according to the severity of weight loss: Non-cachexia group, weight loss ≤ 10 %; Cachexia group, > 10%.

### Assessment of H pylori status

^14^C-UBT test was repeated two times for enrolled patients and controls by a single team of specialized staff. None of the enrolled subjects had ever been treated for H pylori eradication.

### DNA extraction

The genomic DNA was purified from 5 ml of peripheral blood samples using Wizard Genomic DNA Purification kit (Promega) according to the manufacture's instruction.

#### IL-1B+3954 C/T

This polymorphism was investigated using restriction fragment length polymorphism analysis of polymerase chain reaction products as previously studied [16].

#### IL-1B-31 T/C

Forward, TCTTTTCCCCTTTCCTTTAACT; reverse, GAGAGACTCCCTTAGCACCTAGT [17] (Nanjing Bio Eng Co.). PCR conditions: 10 min at 95°C; 40 cycles of 30 sec at 72°C, 15 sec at 95°C, 20 sec at 52°C (BIO-RAD, Japan). PCR products were digested by restriction endonuclease Alul (Promega) and visualized by electrophoresis on a 2.5% agarose gel stained with 0.1% ethidium bromide. Alleles were coded as follows: C allele: 234 bp; T allele: 150 bp and 84 bp.

#### IL-1B-511C/T

Forward, 5-TGGCATTGATCTGGTTCATC-3, reverse, 5-GTTTAGGAATCTTCCCACTT-3 [18] (Nanjing Bio Eng Co.). PCR conditions: 95°C 1 minute; 30 cycles of 95°C 30 sec, 55°C 30 sec, 72°C 30 sec; 70°C 7 min (BIO-RAD, Japan). PCR products were digested by restriction endonuclease Aval (Promega) and visualized by electrophoresis on a 2.5% agarose gel stained with 0.1% ethidium bromide. Alleles were coded as follows: T: 304 bp, C: 190 and 114 bp.

#### IL-1RN VNTR

Forward, 5-CTCAGCAACACTCCTAT-3, reverse, 5-TCCTGGTCTGCAGGTAA-3 [19] (Nanjing Bio Eng Co.). PCR conditions: 94°C for 4 min, 32 cycles of 94°C for 1 min, 50°C for 1 min, 72°C for 1 min ; 72°C for 10 min (BIO-RAD, Japan). PCR products were analyzed by electrophoresis on a 2% agarose gel stained with ethidium bromide. Alleles 1–5 (IL1RN*1-IL1RN*5) were detected according to their sizes relative to a 100 bp DNA ladder: allele 1 (four repeats) 410 bp; allele 2 (two repeats) 240 bp; allele 3 (five repeats) 500 bp; allele 4 (three repeats) 325 bp. Allele 5 (six repeats; 595 bp) was not observed in the study population.

### Statistical analysis

Descriptive data of continuous variables were reported as mean ± SD and tested by Student's *t*-test by SPSS 11.0. The differences in allele frequency among groups were examined for statistical significance with chi-square test or Fisher exact test when appropriate. Analysis was completed by SAS 6.12, and a 2-tailed *P*<0.05 was taken to denote significance.

## Results

### Hardy-Weinberg equilibrium

Within each study group, the genotype distributions were consistent with those predicted by the Hardy-Weinberg equilibrium (table 1 and table 2.).

### Characteristics of the study population

On the basis of the selection criteria, 214 patients with locally advanced gastric cancer were studied. Of these, 91 patients had a greater than 10% weight loss within a six-month period (cachexia group). The characteristics of the study population were shown in table 3. No significant difference was noted in sex, age, tumor stage and locations between patients with cachexia and those without.

The clinical characteristics of the controls and the patients were shown in table 4.

### Gene polymorphisms in patients with locally advanced gastric cancer and controls

No significant difference was found in allele frequency between patients and controls (Table 5).

### Distribution of alleles studied in patients with locally advanced gastric cancer

Details of the comparison of alleles in patients with gastric cancer are shown in table 6. No significant differences were found in the distributions of IL-1-31 T, -511 T and IL-1RN VNTR allele between cachexia group and non-cachexia group.

As to IL-1B+3594 polymorphism, IL-1B+3594 T allele was seen significantly more frequently in the patients with cachexia than without (χ^2 ^= 5.556, *P *= 0.018).

### Logistic regression

Logistic regression adjusted for actual weight, carcinoma location and stage, the IL-1β+3954 CT genotype was associated with an odds ratio of 2.512 (95% CI, 1.180 – 5.347) for cachexia (table 7).

## Discussion

In view of the inflammatory nature of cancer cachexia, we explored the role of polymorphisms in genes related to inflammation in gastric cancer. The frequency of IL-1B-31 T, -511 T, +3954 T and IL-1RN VNTR alleles found in this study is comparable to the previous reports in China [20-22]. We found that polymorphisms studied are not associated with a significantly increased risk of locally advanced gastric cancer. However, a significantly increased frequency of IL-1B+3954 T allele was noted in patients with cachexia from locally advanced gastric cancer, which has not been reported previously.

Before evaluating the role of a cytokine polymorphism played in a disease, three questions need to be answered [23,24]. First, are the subjects homogeneous? To avoid artifact in population admixture, we selected only Chinese Han people in China. In addition, the consanguineous mating subjects were precluded from our study. Second, does the product of the studied gene play an important role in the pathogenesis of the disease? The central roles for IL-1β in the occurrence of cachexia have been demonstrated by many studies [3,5,25]. Third, does the gene polymorphism produce a relevant alteration in the level or function of the gene product? In vitro and in vivo studies have indicated that these alleles enhance IL-1β and IL-1RN productions and the circulating levels of the two cytokines in humans [10,11].

Since El-Omar et al [26] reported that IL-1B-31CC and IL-1RN*2/*2 genotypes were associated with a significantly increased risk of GC in Caucasians, some contradictory results from studies in different countries have been reported. Therefore, before evaluating the role of a cytokine polymorphism played in cachexia associated with gastric cancer, one important question need to be answered. Are the gene polymorphisms studied correlated with gastric cancer? In the present study, we investigated the roles of IL-1B-31 T/C, -511 C/T, +3954 C/T and IL-1RN VNTR as host risk factors for gastric cancer, but failed to demonstrate an association between polymorphisms studied and gastric cancer susceptibility. Susceptibility to gastric cancer in patients with proinflammatory genetic profile is a fascinating hypothesis that has been mainly confirmed in studies from Poland [26], Portugal [27,28], and the America [29]. In contrast, other studies performed in Asian failed to confirm this finding [20,30,31]. Although there is no clear explanation for these conflicting results, the different genetic background of Western and Asian populations could be an important factor. In addition, gastric cancer is a multifactorial disease. The marked geographic variation, time trends, and the migratory effect on gastric cancer incidence suggest that environmental or lifestyle factors are major contributors to the etiology of this disease. Thus, it is necessary to perform large-scale case-control studies considering lifestyle factors such as diet and drinking habits before a final statement of the role of IL-1B gene polymorphism in gastric cancer can be made. However, our present study from this Chinese population provides evidence that no association was seen between polymorphisms studied and gastric cancer, and thus no evidence that these loci contribute to advanced gastric cancer susceptibility.

While the precise mechanism of cachexia is unclear it is self evident that the inflammatory process is exceedingly strong in weight losing cancer patients [32]. In this study, we examined the cytokine polymorphism frequencies in the patients with locally advanced gastric. However, the distribution of cytokine polymorphisms within the patients varied, with IL-1B+3954 T allele being found significantly more frequently in the cachexia patients than in non-cachexia patients (*P *< 0.05). Logistic regression adjusted for actual weight, carcinoma location and stage, the IL-1β+3954 CT genotype was associated with an odds ratio of 2.512 (95% CI, 1.180 – 5.347) for cachexia. In vitro study demonstrated that IL-1β+3594 T at the 5th exon significantly influences the production of IL-1β [10]. Therefore, we induce that gene-controlled high production of IL-1β are likely to play some role in the occurrence of cachexia from patients with advanced gastric cancer. However, in studies with a relatively small sample size, such as the present study, attention should be paid to the evaluation of significant factors suggested by statistical analysis, especially when they have relatively large P values (0.01 <*P *< 0.05). There is a possibility that such factors show a false-positive result.

There are several strengths of our gene association study. First, patients were recruited from two centers; the number of patients is relatively big compared with studies with smaller sample size. Second, to avoid artifact in population admixture, we selected only Chinese Han people in China, thus limiting the chance of population stratification due to ethnic. Third, we studied four separate but related genes, which allows direct comparison of the results of each gene polymorphism association with outcome. However, the interpretation of our study results is limited because it might be more valuable to measure the actual level or to assess the expressions of IL-1β in the gastric mucosa according to the polymorphisms. In addition, we didn't evaluate the protein loss of the subjects because such factor was difficult to be expressed numerically and to be evaluated objectively. Doubtless, further studies will identify whether the product of the studied genes play an important role in the pathogenesis of cachexia from advanced gastric cancer.

## Conclusion

In summary, the present study from this Chinese population provides evidence that IL-1β allele may contribute to the occurrence of cachexia associated with locally advanced gastric cancer. Studies with large sample sizes and multiple SNPs of other important cytokines are needed to further elucidate the genetic factors involved in the etiology of cachexia from gastric cancer.

## Competing interests

The author(s) declare that they have no competing interests.

## Authors' contributions

DZ, HZ, YZ, XT, BY and JL were all involved in designing the study. DZ surveyed the patients, HZ, YZ and XT analyzed the data. All authors reviewed the data and were involved in final analysis and conclusions. DZ wrote the first draft of the manuscript to which all authors subsequently contributed. All authors read and approved the final manuscript.

## Pre-publication history

The pre-publication history for this paper can be accessed here:



## Figures and Tables

**Table 1:** Hardy-Weinberg equilibrium analysis in patients with cancer and controls.

		Cancer (n = 214)		Contr. (n = 230)	
		Observed	Expected	Observed	Expected
IL-1β-31	CC	48	45.3	53	49.8
	CT	101	95.1	108	114.4
	TT	75	73.6	69	65.8
*P*		0.980		0.835	
IL-1β-511	CC	55	50.1	56	49.3
	CT	97	106.8	101	114.4
	TT	62	57.1	73	66.3
*P*		0.638		0.454	
IL-1β+3594	CC	178	179.5	201	201.9
	CT	36	33	29	27.2
	TT	0	1.5	0	0.9
*P*		0.442		0.619	
IL-1RN VNTR	1/1	157	156.5	168	167
	1/2	45	54	50	58.8
	2/2	5	3.5	6	4.2
	1/3	5	0	4	0
	1/4	2	0	2	0
*P*		0.089		0.135	

Genotypes did not deviate from the Hardy – Weinberg equilibrium.

**Table 2:** Hardy-Weinberg equilibrium analysis in Non-cachexia group and Cachexia group.

		Non-cachexia (n = 123)		Cachexia (n = 91)	
		Observed	Expected	Observed	Expected
IL-1β-31	CC	26	24.1	12	12.7
	CT	57	60.7	44	42.6
	TT	40	38.1	35	35.7
*P*		0. 889		0.976	
IL-1β-511	CC	31	27.8	24	22.3
	CT	55	61.4	42	45.4
	TT	37	33.8	25	23.3
*P*		0.716		0.881	
IL-1β+3594	CC	109	109.4	69	70.3
	CT	14	13.2	22	19.4
	TT	0	0.4	0	1.3
*P*		0.809		0.480	
IL-1RN VNTR	1/1	91	90.5	66	66.0
	1/2	25	30.5	20	23.4
	2/2	3	2.0	2	1.6
	1/3	3	0.0	2	0.0
	1/4	1	0.0	1	0.0
*P*		0.316		0.510	

Genotypes did not deviate from the Hardy – Weinberg equilibrium.

**Table 3:** Characteristics of the patients with locally advanced gastric cancer.

		Cachexia (n = 91)	Non-cachexia (n = 123)	*P*
Sex (M/F)		55/36	72/51	0.7793
Age (year)		55.5 ± 15.0	57.0 ± 13.5	0.4443
Serum albumin (g/L)		30.5 ± 4.7	36.3 ± 6.1	<0.001
Actual weight (kg)		55.1 ± 9.0	57.2 ± 6.3	0.0584
Anorexia (n)		39	46	0.4198
Carcinoma location	Middle third	56	90	0.071
	Low third	35	33	
Carcinoma stage	III	65	101	0.064
	IV	26	22	

No significant difference was noted in sex and age.

**Table 4:** Characteristics of the patients and controls.

		Cancer (n = 214)	Contr. (n = 230)	*P*
Sex (M/F)		127/87	140/90	0.743
Age (year)		56.4 ± 14.3	55.0 ± 13.5	0.280
Smoking status	never	85 (39.7%)	95 (41.3%)	0.714
	Ex-	32 (15.0%)	39 (17.0%)	
	current	97 (45.3%)	96 (41.7%)	
Helicobacter pylori infection		125 (58.4%)	*124 (59.9%)	0.756
Poor drinking water (well water)		31 (14.5%)	22 (9.6%)	0.110

* Twenty-three controls missed. No significant association was found between the frequency of smoking status, poor drinking water status and Helicobacter pylori infection. The two groups were matched for age and sex.

**Table 5:** Comparison of alleles between patients with cancer and controls.

		Cancer (n = 214)	Contr. (n = 230)	*P*
IL-1β-31	CC	48	53	
	CT	101	108	
	TT	75	69	
	T allele	251(58.6 %)	246(53.5%)	0.121
IL-1β-511	CC	55	56	
	CT	97	101	
	TT	62	73	
	T allele	221(51.6%)	237 (53.7%)	0.973
IL-1β+3594	CC	178	201	
	CT	36	29	
	TT	0	0	
	T allele	36 (8.4 %)	28 (6.3%)	0.228
IL-1RN VNTR	1/1	157	168	
	1/2	45	50	
	2/2	5	6	
	1/3	5	4	
	1/4	2	2	0.992

No significant difference was found in allele frequency between patients with cancer and controls

**Table 6:** Distribution of alleles in the patients with cancer.

		Non-cachexia (n = 123)	Cachexia (n = 91)	*P*
IL-1β-31	CC	26	12	
	CT	57	44	
	TT	40	35	
	T allele	137 (55.7 %)	114(62.6 %)	0.194
IL-1β-511	CC	31	24	
	CT	55	42	
	TT	37	25	
	T allele	109 (44.3 %)	92 (50.5%)	0.376
IL-1β+3594	CC	109	69	
	CT	14	22	
	TT	0	0	
	T allele	14 (5.7 %)	22 (12.1 %)	0.018
IL-1RN VNTR	1/1	91	66	
	1/2	25	20	
	2/2	3	2	
	1/3	3	2	
	1/4	1	1	0.997

*χ^2 ^= 5.556,*P *= 0.018. Significant difference was found in T allele frequency between cachexia group and non-cachexia group.

**Table 7:** Logistic Regression Analysis in patients with gastric caner.

	β	95% C.I	S.E	P
IL-1β +3954	0.921	2.512(1.180, 5.347)	0.386	0.017

Binary Logistic regression. Method = Stepwise (Conditional). Logistic regression adjusted for actual weight, carcinoma location and stage, the IL-1β +3954 CT genotype was associated with an odds ratio of 2.512 (95% CI, 1.180 – 5.347) for cachexia
